# Spatiotemporal oscillations of Notch1, Dll1 and NICD are coordinated across the mouse PSM

**DOI:** 10.1242/dev.115535

**Published:** 2014-12-15

**Authors:** Robert A. Bone, Charlotte S. L. Bailey, Guy Wiedermann, Zoltan Ferjentsik, Paul L. Appleton, Philip J. Murray, Miguel Maroto, J. Kim Dale

**Affiliations:** 1 Division of Cell and Developmental Biology, College of Life Sciences, University of Dundee, Dow Street, Dundee DD1 5EH, UK; 2 School of Biology, University of Nottingham, University Park, Nottingham NG7 2RD, UK; 3 Division of Mathematics, University of Dundee, Dow Street, Dundee DD1 5EH, UK

**Keywords:** Notch signalling, Oscillations, Somitogenesis

## Abstract

During somitogenesis, epithelial somites form from the pre-somitic mesoderm (PSM) in a periodic manner. This periodicity is regulated by a molecular oscillator, known as the ‘segmentation clock’, that is characterised by an oscillatory pattern of gene expression that sweeps the PSM in a caudal-rostral direction. Key components of the segmentation clock are intracellular components of the Notch, Wnt and FGF pathways, and it is widely accepted that intracellular negative-feedback loops regulate oscillatory gene expression. However, an open question in the field is how intracellular oscillations are coordinated, in the form of spatiotemporal waves of expression, across the PSM. In this study, we provide a potential mechanism for this process. We show at the mRNA level that the *Notch1* receptor and Delta-like 1 (*Dll1*) ligand vary dynamically across the PSM of both chick and mouse. Remarkably, we also demonstrate similar dynamics at the protein level; hence, the pathway components that mediate intercellular coupling themselves exhibit oscillatory dynamics. Moreover, we quantify the dynamic expression patterns of Dll1 and Notch1, and show they are highly correlated with the expression patterns of two known clock components [*Lfng* mRNA and the activated form of the Notch receptor (cleaved Notch intracellular domain, NICD)]. Lastly, we show that *Notch1* is a target of Notch signalling, whereas *Dll1* is Wnt regulated. Regulation of *Dll1* and *Notch1* expression thus links the activity of Wnt and Notch, the two main signalling pathways driving the clock.

## INTRODUCTION

During somitogenesis, epithelial spheres called somites bud off from the most rostral end of the pre-somitic mesoderm (PSM) in a rostral-to-caudal direction and, later in development, these give rise to the vertebral column, most of the skeletal musculature and much of the dermis (Dequeant and Pourquie, 2008). This occurs with a remarkable periodicity that is regulated by a molecular oscillator (Cooke and Zeeman, 1976), which drives cyclic waves of gene expression caudo-rostrally through the PSM with the same periodicity as that of somite formation. This periodicity is species specific; in chick it is 90 min, in mouse 120 min and in humans 4-5 h (Dequeant and Pourquie, 2008). The majority of known clock genes belong to the Notch pathway (reviewed by Dequéant and Pourquié, 2008; Gibb et al., 2010; Kageyama et al., 2007) and, in mouse, this pathway is crucial for dynamic expression of all clock genes and for somitogenesis (Ferjentsik et al., 2009). A number of Wnt and FGF pathway members also cycle in the mouse PSM (Dequeant and Pourquie, 2008). Furthermore, these three pathways interact reciprocally within the mechanism of the mouse segmentation clock (Dequeant and Pourquie, 2008; Gibb et al., 2010; Maroto et al., 2012; Niwa et al., 2007). It is widely accepted, on a single cell level in the vertebrate PSM, that oscillatory gene expression is established through negative-feedback loops of unstable clock gene products (Hirata et al., 2004; Lewis, 2003; Monk, 2003). Thus, in the case of the Notch pathway, it seems relatively clear how intracellular negative-feedback loops, involving Lfng and Hes7 proteins, contribute to the regulation of Notch target gene expression in a cell-autonomous manner.

In contrast to the intracellular picture, there is no well-established biological model describing the mechanism by which neighbouring cells coordinate and co-regulate spatiotemporal oscillations across the PSM. This point is particularly significant as, although recent theoretical and experimental studies (Murray et al., 2011; Lauschke et al., 2013) have highlighted a fundamental role for phase differences between neighbouring oscillators in the emergence of spatiotemporal gene expression patterns in the PSM, the crucial question of precisely how phase differences are communicated between neighbouring cells remains unresolved. Cell-cell contact appears to be essential (Maroto et al., 2005), and what is known about intercellular coupling in the PSM is that Notch signalling is likely to play a fundamental role (Jiang et al., 2000). For instance, live imaging in the zebrafish PSM indicates that both local coordination and tissue-scale waves of clock gene expression are lost in Notch mutants (DeLaune et al., 2012). Moreover, artificial pulses of expression of the Notch ligand DeltaC restore synchrony and rescue somite formation in these mutants (Soza-Ried et al., 2014). These data suggest that the entrainment of intracellular oscillations is mediated by periodic activation of the Notch pathway. Furthermore, we note that a key event in the canonical Notch signalling pathway is the release, following ligand activation, of Notch intracellular domain (NICD) and its subsequent transport to the nucleus, where it activates a range of Notch pathway targets. In the mouse PSM it has been established that NICD exhibits pulsatile spatiotemporal waves of expression that traverse the rostro-caudal axis in the manner of a clock gene (Huppert et al., 2005). However, it is not well understood how this pattern emerges, as it has been widely reported that both Dll1 and Notch1 proteins are expressed in a rostro-caudal gradient in the PSM (Chapman et al., 2011; Sparrow et al., 2012; Okubo et al., 2012).

In this study, we show in both chick and mouse that *Dll1* and *Notch1* oscillate across the PSM (at the mRNA level) in a Wnt- and Notch1-dependent manner, respectively. Our data show that as well as exhibiting rostro-caudal gradients, Dll1 and Notch1 protein levels undergo spatiotemporal oscillations in the PSM that are coordinated with *Lfng* pre-mRNA and NICD oscillations. Taken together, these novel data indicate a mechanism by which inter-cell phase differences can be communicated between neighbouring cells; oscillatory levels of functional signalling components enable pulsatile activation of the pathway.

## RESULTS

### *Dll1* and *Notch1* mRNA expression is dynamic across the mouse PSM

**Fig. 1.**
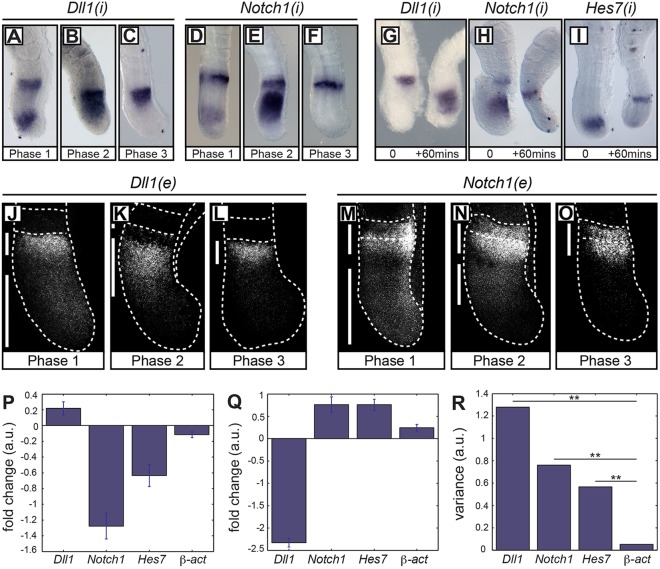
***Dll1* and *Notch1* mRNA expression in mouse PSM.** (A-F) *In situ* hybridisation of *Dll1(i)* (A-C) and *Notch1(i)* (D-F) in E10.5 PSM using intronic *(i)* RNA probes. (G-I) Fix-and-culture assay comparing the expression pattern of *Dll1(i)* (G) and *Notch1(i)* (H) with that of the clock gene *Hes7* (I). (J-O) FISH of *Dll1* (J-L) and *Notch1* (M-O) mRNA in PSM using exonic *(e)* probes. (P-R) qRT-PCR for *Dll1*, *Notch1*, *Hes7*, β-actin and *Gapdh* in the caudal halves of individual E10.5 PSM explants following fix and culture. Data show the mean ± s.d. of technical replicates. (P,Q) Individual samples showing the fold change in mRNA concentration between fixed and cultured explants for *Dll1*, *Notch1*, *Hes7* and β-actin once normalised to *Gapdh*. (R) Total variance of fold change in expression levels in fix:culture ratios of each gene measured by qRT-PCR across all 16 samples. a.u., arbitrary units. **P<0.001.

To investigate whether the expression of Dll1 and Notch1 displays an oscillatory pattern in the PSM, we initially examined nascent *Dll1* and *Notch1* mRNA expression [using *in situ* hybridisation with antisense probes that hybridise to intronic regions of these nascent transcripts, which are referred to here as *Dll1(i)* and *Notch1(i)*]. We found that *Dll1(i) and Notch1(i)* PSM expression varied spatially across different embryonic day (E)10.5 mouse embryos [*n*=48 and *n*=25, respectively; Fig. 1A-F; 15 Phase 1, 20 Phase 2 and 13 Phase 3 embryos for *Dll1(i)*; 6 Phase 1, 10 Phase 2 and 9 Phase 3 embryos for *Notch1(i)*]. To rigorously demonstrate that these variations are due to dynamic gene expression we used the ‘fix and culture’ assay (see Materials and Methods). Expression of both *Dll1(i) and Notch1(i)* were different in the two halves of each embryo analysed with this assay, thereby confirming dynamic activity (*n*=5/5 and *n*=7/7, respectively; Fig. 1G-I). Thus, nascent *Dll1* and *Notch1* mRNA expression oscillates in the PSM in the manner of a clock gene and their transcription is therefore likely to be regulated by the segmentation clock.

Numerous reports have previously described the expression of mature *Dll1* and *Notch1* mRNA as a static rostro-caudal gradient in the PSM, observations that are seemingly at odds with our data describing dynamic expression of nascent transcripts across the PSM for these genes. In order to investigate this issue, we used two approaches that are more sensitive than those used in the previous analyses – fluorescent *in situ* hybridization (FISH) and quantitative reverse-transcription PCR (qRT-PCR). Using FISH, we found that the expression of mature *Dll1* and *Notch 1* mRNA varies considerably across different E10.5 embryos (*n*=19 and *n*=23, respectively; Fig. 1J-O; 10 Phase 1, 7 Phase 2 and 2 Phase 3 embryos for *Dll1*; 4 Phase 1, 13 Phase 2 and 6 Phase 3 embryos for *Notch 1*), in line with findings for Delta1 by Maruhashi et al. (2005). Furthermore, by using qRT-PCR in the E10.5 caudal PSM, we observed and quantified clear changes in expression levels between fixed and cultured explants within individual embryos (Fig. 1P,Q; *n*=16). In order to ascertain whether changes in *Dll1* and *Notch 1* expression exhibited a discernable pattern across all measured embryos, we calculated the total variance for each gene, across all fix and culture explant pairs using analysis of variance (R Core Team, 2013; http://www.R-project.org/; see supplementary material Table S4) and found highly statistically significant changes in *Dll1*, *Notch 1* and *Hes7* expression compared with those of the housekeeping genes *β-actin* and *Gapdh* (Fig. 1R; see supplementary material Table S5). We note that expression of both nascent and mature *Dll3* mRNA, the only other Notch ligand broadly expressed in the PSM, is non-dynamic (data not shown; *n*=10 and 15, respectively). Taken together, these data provide strong evidence that both nascent and mature *Dll1* and *Notch1* mRNA undergo oscillatory dynamics in the caudal mouse PSM.

### *Dll1* and *Notch1* nascent mRNA transcription is dynamic across the chick PSM

**Fig. 2.**
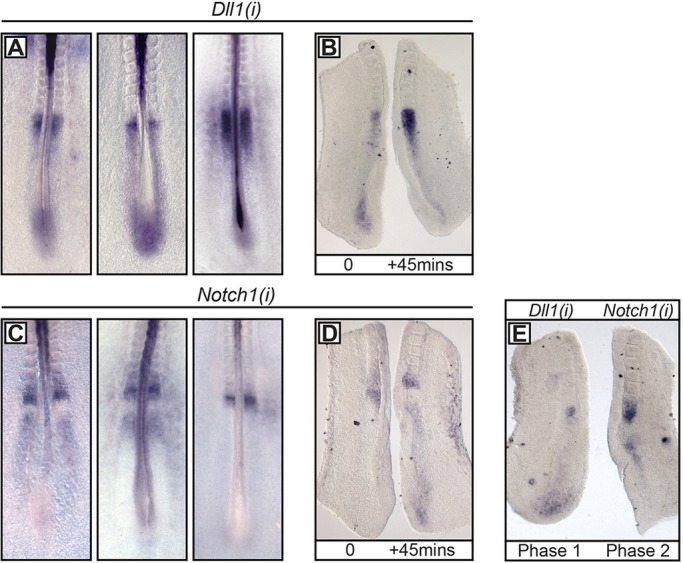
**Pre-mRNA expression profile of *Dll1* and *Notch1* in the chick PSM.** (A-E) *In situ* hybridisation of *Dll1(i)* and *Notch1(i)* in the PSM of HH8-HH13 embryos using intronic *(i)* RNA probes. Cyclical expression was confirmed by fix-and-culture analysis for *Dll1(i)* (B) and *Notch1(i)* (D). (E) *Dll1(i)* and *Notch1(i)* oscillate out of synchrony.

Given that the levels of *DeltaC* mRNA oscillate in the zebrafish PSM, we investigated whether it is a conserved feature of the segmentation clock. We found that the PSM expression of both *Dll1* and *Notch1* nascent mRNA varied considerably in Hamburger–Hamilton (HH) stage 8-13 embryos (Fig. 2A,C, respectively; *n*=21), and we confirmed cyclical expression by performing the fix-and-culture assay (Fig. 2B,D; *n*=22/27). Thus, *cDll1* and *cNotch1* nascent mRNA expression also exhibits oscillatory patterns across the chick PSM.

**Fig. 3.**
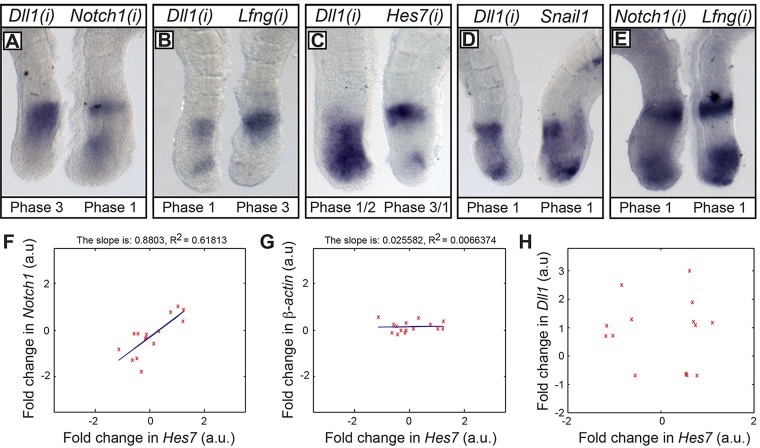
**Comparison of *Dll1* and *Notch1* mRNA expression with that of clock genes.** (A-E) *Dll1(i)* oscillates out of synchrony with *Notch1(i)* (A), *Lfng(i)* (B) and *Hes7(i)* (C). *Dll1(i)* oscillates in synchrony with *Snail1* (D). *Notch1(i)* oscillates in synchrony with *Lfng(i)* (E). (F-H) Fold changes (normalised to *Gapdh*) between E10.5 caudal PSM fixed and cultured samples, as determined by using qRT-PCR, for *Notch1* against *Hes7* (F), β-actin against *Hes7* (G) and *Dll1* against *Hes7* (H). a.u., arbitrary units.

To compare *Dll1* and *Notch1* expression in the same embryo, we used the ‘half embryo’ assay, whereby the two half explants are hybridised with a probe to detect a different gene. The two genes oscillated out of phase in both mouse and chick (*n*=6/8, *n*=13/18, respectively; Fig. 3A, Fig. 2E). This result suggests that the expression of *Dll1* and *Notch1* in the PSM is regulated by different signalling activities.

### *Dll1(i)* transcription cycles in phase with Wnt clock genes, whereas *Notch1(i)* cycles in phase with Notch clock genes

Wnt regulates *Dll1* expression in the mouse PSM (Galceran et al., 2004; Hofmann et al., 2004), whereas Notch regulates dynamic *DeltaC* expression in the zebrafish PSM (Jiang et al., 2000). To ascertain which pathway regulates dynamic expression of *Dll1(i)*, we compared its expression with that of known Notch and Wnt cyclic targets in contralateral halves of an embryo. The results showed that *Dll1(i)* oscillates out of phase with the Notch targets *Lfng(i)* (*n*=6/8) and *Hes7(i)* (*n*=14/15; Fig. 3B,C). *Dll1(i)* expression oscillates largely in synchrony with the Wnt target *Snail1* (Fig. 3D; *n*=10/15; Dale et al., 2006). In contrast, *Notch1(i)* cycles in phase with the Notch target *Lfng(i)* (Fig. 3E; 9/12).

It is notable that these *in situ* nascent mRNA data are consistent with those from the previously described qRT-PCR experiments. Moreover, when fold changes for *Notch1* and *Hes7* mRNA are plotted relative to each other for all samples analysed, there is a highly significant positive correlation (*F*_1,13_=21.04; *P*≤0.001), which is not the case for *Dll1* and *Hes7* or for *Dll1* and *Notch1* (Fig. 3F-H; data not shown).

### *Dll1* and *Notch1* expression are regulated by Wnt and Notch, respectively

**Fig. 4.**
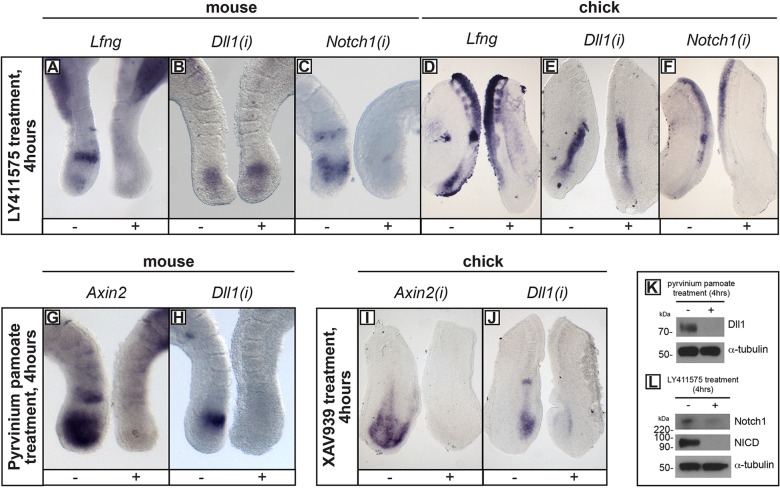
**Notch and Wnt inhibition reveals differing regulation of *Dll1(i)* and *Notch1(i)* expression.** (A-J) *In situ* hybridisation was performed on mouse or chick explants cultured with (+) or without (−) LY411575 (A-F), pyrvinium pamoate (G,H) or XAV939 (I,J). (K,L) Western blot analysis of Dll1 protein from pooled PSM samples following 4 h exposure to pyrvinium pamoate (K), or analysis of Notch1 and NICD proteins following 4 h exposure to LY411575 (L).

We next addressed how the expression of these two genes is regulated in both the mouse and chick PSM. Many clock genes in mouse, fish and chick are Notch targets. In some developmental and disease contexts, Notch expression is itself dependent on Notch signalling (Agrawal et al., 2009; Bray and Bernard, 2010; de Celis and Bray, 1997; Weng et al., 2006). In the context of the PSM this has not been investigated. Explants cultured in the presence of the γ-secretase inhibitor LY411575 (Lanz et al., 2004), used at 150 nM, showed that *Notch1(i)* mRNA expression is abolished in the PSM (Fig. 4C,F; *n*=25/29 mouse; *n*=7/7 chick), as seen for the control, *Lfng* (Fig. 4A,D; *n*=12/12 mouse; *n*=7/7 chick). In contrast, there is no downregulation of *Dll1(i)* expression under these conditions (Fig. 4B,E; *n*=9/12 mouse; *n*=8/8 chick). To determine which pathway regulates dynamic *Dll1(i)* transcription in the mouse and chick PSM, we inhibited Wnt signalling using the Wnt inhibitors pyrvinium pamoate (Thorne et al., 2010) and XAV939 (Huang et al., 2009), respectively. Pyrvinium pamoate (10 µM) inhibits mRNA expression of the Wnt target *Axin2* (Fig. 4G; *n*=7/7) and of *Dll1* (Fig. 4H; *n*=7/7) in the mouse, and XAV939 (100 µM) inhibits their expression in the chick (Fig. 4I,J; *n*=13/14; and 8/10, respectively). These data indicate that dynamic expression of nascent *Dll1* mRNA is Wnt dependent in both mouse and chick. Intriguingly, *Notch1(i)* expression is reduced in some cases following Wnt inhibition (*n*=9/16 mouse; *n*=7/18 chick; data not shown). However, this is not surprising, given that loss of Dll1 will lead to loss of activation of Notch targets, including Notch1 itself, as reported previously (Galceran et al., 2004). Nontoxicity for all treatments was assessed using terminal deoxynucleotidyl transferase dUTP nick end labeling (TUNEL) staining in treated versus control explants (*n*=3 per drug; *n*=9 technical triplicates; *P*>0.05 for all treatments; supplementary Materials and Methods; supplementary material Fig. S1). Western blot analysis revealed that levels of Dll1 and Notch1 proteins were also severely downregulated following exposure to Wnt or Notch inhibition (Fig. 4K,L, respectively). These data indicate that in both mouse and chick, PSM expression of *Dll1* is Wnt dependent, whereas the expression of *Notch1* is Notch dependent.

### Dll1 and Notch1 protein expression is dynamic across the mouse PSM

**Fig. 5.**
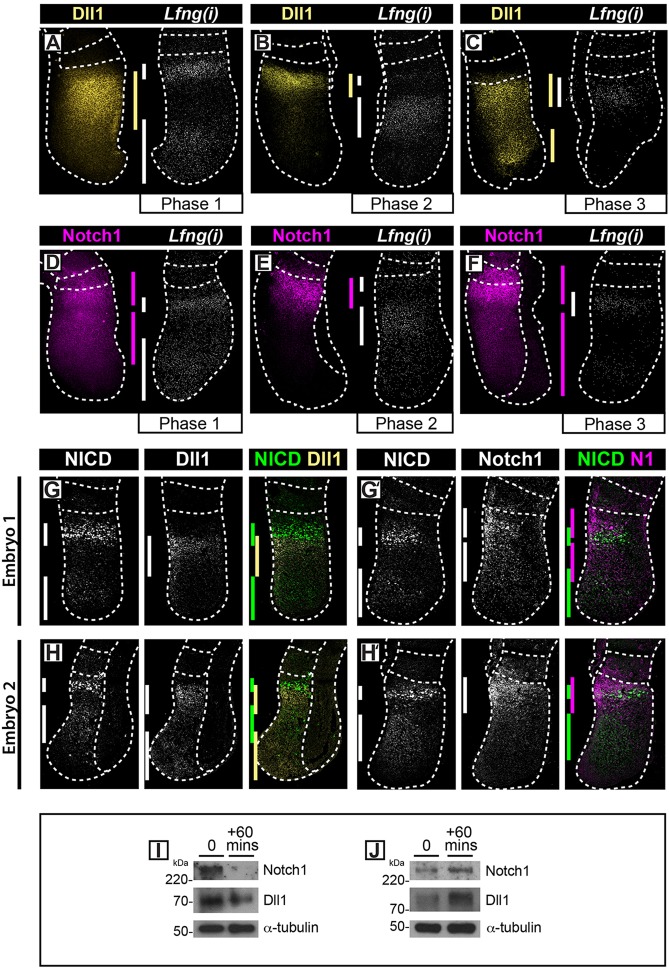
**Oscillations of Dll1 and Notch1 proteins in mouse PSM.** (A-F) Following FISH to detect *Lfng(i)* in one half of a set of E10.5 tails, immunohistochemistry was performed on the contralateral half of the explants to detect Dll1 (A-C) or Notch1 (D-F) protein. (G-H′) Double immunohistochemistry on sections of individual E10.5 tails to detect PSM expression of NICD and Dll1 (G,H), or NICD and Notch1 (G′,H′) (*n*=15). The dotted lines demarcate the positions of the most recently formed somite(s), outer edges of the PSM, adjacent neural tissue (C,E) or hind gut (H). (I,J) Western blot analysis for Dll1 and Notch1 or α-tubulin on the caudal half of individual PSM explants following fix and culture.

Having demonstrated that levels of nascent and mature *Dll1* and *Notch1* transcripts are spatiotemporally regulated in the PSM, we investigated the dynamics of the protein products they encode. To allow analysis of protein expression with respect to a known cycling Notch target within the same embryo, we first compared the expression of Dll1 protein and *Lfng* nascent mRNA in individual embryos using contralateral PSM explants of E10.5 mouse tails. We found that the spatial expression profile of Dll1 protein varied across different mouse tails, while the *Lfng* pre-mRNA expression confirmed that these individual mouse tails were in different phases of the oscillation cycle (*n*=14; Fig. 5A-C). Using this assay, we also found that the spatial expression of Notch1 protein varied across mouse tails that were in different phases of the oscillation cycle (*n*=11; Fig. 5D-F). These data demonstrate that Dll1 and Notch1 protein expression profiles exhibit spatiotemporal variations across different samples.

We repeated this analysis in sectioned tissue, using an alternative internal control cyclic marker, NICD. We compared by immunohistochemistry the NICD expression profile to that of Dll1 or Notch1 protein expression in alternate paraffin sections of the same E10.5 tail. As expected, we observed variation in the spatial patterns of NICD as observed previously (*n*=15; Fig. 5G-H′; Huppert et al., 2005). Notably, we again observed spatial variations in Notch1 and Dll1 expression across the sample set that were consistent with the explant data (Fig. 5G-H′).

**Fig. 6.**
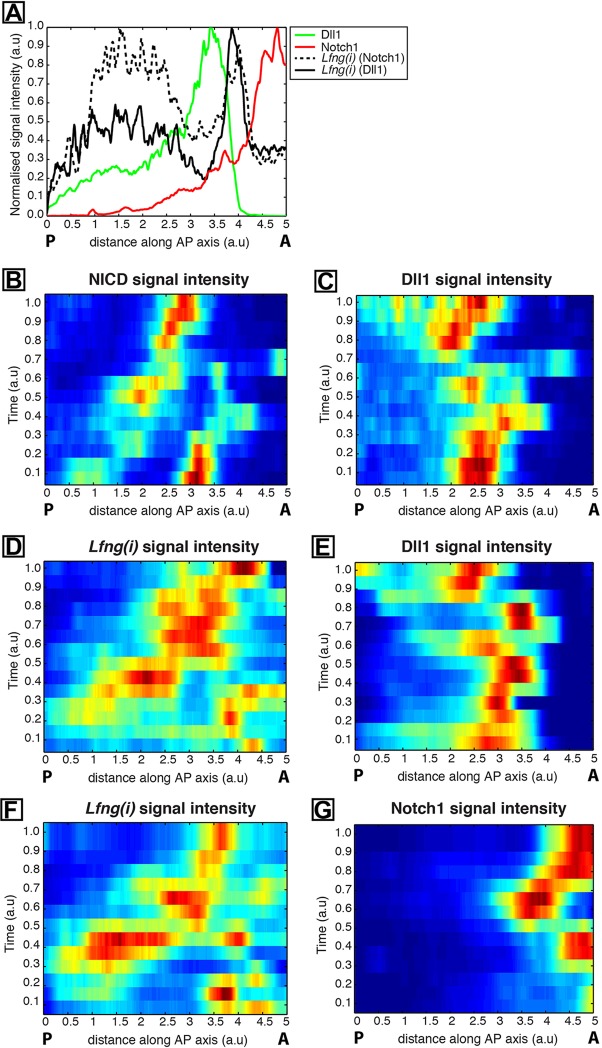
**Quantification of spatiotemporal dynamics of Dll1 and Notch1 protein expression.** (A) An example of an intensity plot depicting axial variation in signal intensity across the PSM. Data plotted from two explant pairs showing *Lfng* pre-mRNA (black broken line) in one explant compared with Notch1 protein (red) in the contralateral half explant, and *Lfng* pre-mRNA (black unbroken line) in a half explant from a second tail compared with Dll1 protein (green) in the contralateral half explant of the second tail. Measured intensities (*y* axis) are plotted against axial position [*x* axis; rostral (‘A’) to right and caudal (‘P’) to left]. (B-H) Kymographs show spatial distribution of Notch1, Dll1 and NICD, and of *Lfng(i)* across numerous PSMs. (B,C) NICD (B) and Dll1 (C) expression in PSM sections; (D,E) *Lfng(i)* (D) and Dll1 (E) in contralateral explant halves; (F,G) *Lfng(i)* (F) and Notch1 (G) in contralateral explant halves. a.u., arbitrary units.

To determine whether our observations of variation in Dll1 and Notch1 protein expression exhibited a discernable global pattern, we depicted their spatiotemporal expression patterns in kymographs (Fig. 6). Each row of a given kymograph represents the expression level plotted as a function of axial position (Fig. 6A). Control kymographs [NICD, *Lfng(i)* and *Lfng(i)*] and their corresponding partners (Dll1, Dll1 and Notch1) are plotted in left- and right-hand columns, respectively (Fig. 6B,D,F and C,E,G, respectively). Notably, time-ordering was established within a given kymograph in an entirely automated fashion by finding the sample ordering that maximised temporal periodicity (see Materials and Methods). It is well established that *Lfng(i)* exhibits rostrally travelling waves of expression in the mouse PSM, so we used the *Lfng(i)* samples as positive controls for time ordering. Given the identified ordering of the control gene, the spatiotemporal expression pattern of the partner kymograph of interest was then blindly determined.

The two kymographs generated from the *Lfng(i)* intensity plots are self-consistent (Fig. 6D,F) and clearly reveal spatiotemporal *Lfng(i)* expression patterns that are similar to those described previously, hence providing validation of the described methodology. The kymographs for Dll1 and Notch1 protein also show a smooth transition of the dynamic expression domain and, strikingly, depict pulses of Dll1 (and of Notch1, albeit to a lesser extent) protein expression levels in the caudal PSM (Fig. 6E,G). We note that the alignment of the two *Lfng(i)* kymographs facilitates direct comparison of the two partners (Dll1 and Notch1), thus allowing us to compare the spatiotemporal dynamics from the different datasets.

We highlight that, although the Dll1 kymographs have been generated using both *Lfng(i)* and NICD data that were taken from explants and sections, respectively, they show strikingly similar spatiotemporal dynamics. Moreover, by aligning the two Dll1 kymographs, we can indirectly link the *Lfng(i)* and NICD kymographs. It is clear that regions of space-time that have high levels of both NICD and *Lfng(i)* are strongly correlated. This is precisely what one would expect, given that NICD is the effector and *Lfng(i)* is a target of Notch signaling, but the kymograph comparison reaches this conclusion independently of this assumption.

**Fig. 7.**
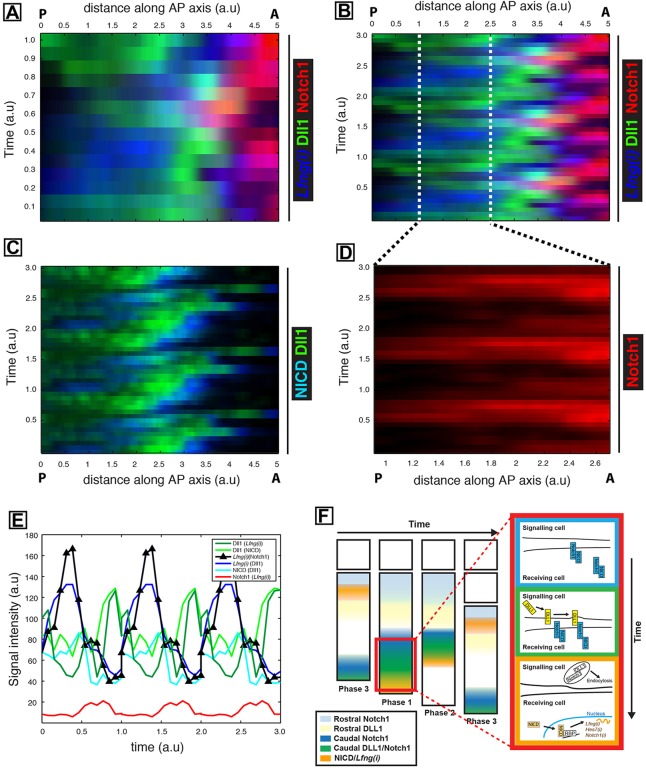
**Quantification of pulsatile Dll1 and Notch1 protein expression in the caudal PSM of mouse tails.** (A) Overlay of kymographs from Fig. 6D [*Lfng(i)*]*,* Fig. 6E (Dll1) and Fig. 6G (Notch1) reveals their spatiotemporal expression during one oscillation cycle. (B) The periodic extension of the data shown in A highlights the oscillatory nature of the dynamics. (C) The periodic extension of Fig. 6B and E depicts regular oscillations and activity waves of both NICD and Dll1 from caudal to rostral that are out of phase with each other. (D) Kymograph to show the intensity of Notch1 alone within the magnified region from B. (E) The average signal intensity for Notch1, Dll1, activated NICD and *Lfng* in caudal regions of the periodically extended kymographs is plotted as a function of time. Note that parentheses in the key denote the corresponding paired sample. Hence, for instance, Dll1 appears twice, as it is paired with both NICD and *Lfng(i)*. a.u. arbitrary units. (F) A proposed model. Pulses of Notch1 protein followed closely by Dll1 originate in the caudal PSM, which initiates a sequence of events detected as spatially separated events along the PSM, depicted as coloured bands of expression. The red boxed region that is magnified to the right contains coloured boxes to show the details of events in each colour band in the PSM. Blue, Notch is translated and translocated to cell membranes; green, Notch 1 and Dll1 are translated and translocate to cell membranes, leading to transactivation; orange, transactivation leads to cleavage and release of NICD, which translocates to the nucleus and activates downstream transcription of Notch target genes (including *Notch1*, *Lfng* and *Hes7*), while Dll1 and/or extracellular Notch1 are endocytosed to be recycled or degraded. Phase 1, 2 and 3 refer to well-established phases of *Lfng*/NICD expression domains.

In order to gain further insight into the spatiotemporal dynamics of Dll1, Notch1 and *Lfng(i)* expression, we overlaid their kymographs. Intriguingly, the spatiotemporal expression of all three factors is tightly coordinated in space and time (Fig. 7A). Extrapolation of the data (Fig. 7A) to multiple cycles of the oscillator in the PSM (Fig. 7B) shows that cells experience pulsatile production (and decay) of Dll1 protein followed by Notch target gene expression as they become rostrally displaced in the PSM (Fig. 7B).

An overlay of the Dll1 and NICD kymographs from the sectioned samples revealed that the two proteins are dynamically expressed and are largely out of phase with one another (see periodically extended kymograph in Fig. 7C). This spatiotemporal separation of Dll1 and NICD profiles suggests that dynamic pulses of Dll1 expression precede the activation of Notch signalling revealed through NICD production.

Curiously, given our observations of oscillatory caudal *Notch1* mRNA expression, Notch1 protein expression is largely obscured in regions of the presented kymographs that represent the caudal PSM. We hypothesised that this arises as a result of the large rostro-caudal gradient in Notch1, which makes the detection and visualisation of the relatively lower levels of Notch1 expression in the caudal PSM challenging. In Fig. 7D, we present a kymograph that displays Notch1 expression data from a restricted caudal region of the PSM, thus obscuring the rostral signal. Strikingly, we observe clear and robust pulsatile profile in this domain.

In order to bring each of the quantities depicted in the kymographs [i.e. Dll1, Notch1, NICD and *Lfng(i)*] together, we calculated, as a function of time, the average signal intensity in caudal regions of the periodically extended kymographs. This exercise revealed that all four factors show dynamic expression in this domain. Moreover, the peak in Dll1 preceeds that of NICD (Fig. 7C) and *Lfng(i)*, which appear in phase with each other (Fig. 7E), whereas Notch1 signal intensity is lower but nevertheless exhibits clear fluctuations that are out of phase with the other components (See Fig. 7E,F).

Finally, to quantitatively verify their cyclic caudal PSM expression, we measured levels of Dll1 and Notch1 protein in the caudal PSM of explants from the same tail by using a fix-and-culture assay followed by western blotting. We observed clear changes in Dll1 and Notch1 protein levels between fixed and cultured explants (Fig. 5I,J; *n*=8). Thus the western blot data independently support the hypothesis that absolute levels of Dll1 and Notch1 undergo temporal oscillations in the caudal PSM. These data provide further corroborative quantitative evidence of the dynamic expression of these proteins within caudal PSM cells.

## DISCUSSION

We provide evidence that transcriptional activation of both the Notch1 receptor and the Dll1 ligand is a cyclical event that occurs in both the chick and mouse PSM. Remarkably, we show that Dll1 and Notch1 proteins also display dynamic expression across the mouse PSM, and that these oscillations are tightly coordinated with the dynamic expression of *Lfng* pre-mRNA and NICD. These results provide the first indication of how highly regulated and dynamic Notch-based intercellular communication might facilitate cell-cell communication and the propagation and synchronisation of the well-established intracellular clock gene oscillations.

We show that *Dll1* mRNA expression in the PSM of both chick and mouse is cyclical. This behaviour does not appear to be general among Delta-like genes, as we did not observe the same dynamism in *Dll3* mRNA expression. In mouse, *Dll1(i)* expression occurs in synchrony with that of the Wnt target *Snail1* and out of synchrony with the expression of Notch-related clock genes. Accordingly, we show that *Dll1* expression in both chick and mouse is lost following Wnt inhibition (Galceran et al., 2004; Hofmann et al., 2004). These data clearly highlight a potentially important point of crosstalk between the Notch and Wnt pathways in the regulation of the segmentation clock mechanism. Although there is some divergence of the specific Notch/Wnt targets that oscillate in different species (Gibb et al., 2009; Dale et al., 2006; Krol et al., 2011), our data highlight a striking degree of conservation of *Dll1* expression in the PSM among vertebrates, given that *DeltaC* oscillates in the zebrafish PSM (Jiang et al., 2000), as does *Dll1* in the PSM of the *Anolis* lizard (Eckalbar et al., 2012). We also show that transcripts for the Notch1 receptor are produced in a pulsatile manner in the mouse and chick PSM that is both synchronous with other Notch-related clock genes and is Notch dependent.

A compelling finding presented in this study is that the expression profiles of Dll1 and Notch1 proteins are also dynamic across the mouse PSM. This highlights a hitherto unknown level of dynamism of clock components in the mouse PSM. It was recently shown that providing artificial pulses of DeltaC can rescue somite defects in Notch mutants (Soza-Ried et al., 2014). Nevertheless, the literature lacked evidence of this pulsatile Dll1 and Notch production at endogenous levels. Our findings provide the first evidence of endogenous pulsatile production of both the Notch1 receptor and the Dll1 ligand in the mouse PSM, which is likely to drive the pulsatile activation of the Notch pathway, seen through NICD expression.

Quantification of dynamic Dll1/Notch1 protein expression patterns and direct comparison with those of *Lfng* pre-RNA or NICD reveals a number of important findings that give mechanistic insight into the emergence of spatiotemporal gene expression patterns in the PSM. First, kymographs, generated from multiple embryos, reveal that the spatiotemporal dynamics of Dll1 and Notch1, measured over the course of one oscillation of the somitogenesis clock, are tightly correlated with those of *Lfng* pre-RNA. Second, combining different datasets in overlaid kymographs has yielded insight into how the oscillations of full-length Dll1 and Notch1 proteins, activated NICD and a read-out of Notch signalling (nascent *Lfng* mRNA) are co-regulated along the rostro-caudal PSM axis. Third, the dynamic spatiotemporal expression of Dll1 and Notch1 provides a potential mechanistic explanation for how the Delta-Notch signalling pathway mediates intercellular coupling, both locally, to coordinate clock gene expression among neighbouring PSM cells, and to coordinate dynamic clock gene expression at the tissue scale across the PSM. Finally, these data provide a crucial missing link to recent studies that highlighted the fundamental role played by phase differences in the emergence of spatiotemporal oscillations (Murray et al., 2013; Lauschke et al., 2013), by providing a mechanism through which these phase differences could be communicated between neighbouring cells.

It is noteworthy that Notch1 protein expression and its message appear to be out of phase, whereas this does not appear to be the case with Dll1. Recent data suggest that delays in the production, splicing, export and translation of transcripts for clock genes are required to generate the oscillatory profile of these factors (Hoyle and Ish-Horowicz, 2013). It is likely that these delay kinetics regulating transcription, nuclear export and/or translation are different for differently sized genes (*Notch1* has 33 introns, 34 exons and a transcript length of 9488 bp, whereas *Dll1* has 10 introns, 11 exons and a transcript length of 3581 bp). Evaluation of the relative weight of contribution of each of these steps in setting the time taken from production of nascent mRNA to that of protein for genes of different lengths will require further investigation.

Previous reports describing Dll1 and Notch1 expression along the PSM have only detected a rostro-caudal gradient of expression (Chapman et al., 2011; Sparrow et al., 2012; Okubo et al., 2012). We used a variety of quantitative techniques, on both optical and paraffin sections of PSM tissue, to analyse changes in intensity of signal in the whole PSM, but also focusing specifically in the caudal PSM. Our data support the idea that there is a gradient of Dll1 and Notch1 expression in the rostral PSM (clearly visible from the kymographs), but we are also able to detect clear oscillations of protein expression in the caudal PSM, albeit at much lower levels than the rostral region in the case of Notch1. One potential explanation for the discrepancies between the observations described by Chapman et al. and those described here is that Chapman and colleagues used unfixed tissue. By fixing the tissue it might be that areas in which Dll1 and Notch1 expression is more transitory (in the caudal PSM) have been more thoroughly preserved, while increased permeabilisation might have allowed greater access for the primary antibodies to bind to Dll1 and Notch1 proteins within the cells of the PSM. Hence, increased sensitivity of signal detection of Dll1 and Notch1 might have been achieved using the methodology we have described.

Having established that there is dynamic expression of Dll1 and Notch1 protein in the caudal PSM, we devised an algorithm to entirely automate and blindly determine the time-ordering of samples, which reveals striking oscillatory patterns of Dll1 and Notch1 and shows that their peaks are coordinated across the PSM tissue. We find that peaks of NICD occur immediately following Dll1 protein peaks, suggesting that dynamic pulses of Dll1 expression precede activation of Notch signalling. The coincidence of *Lfng(i)* and NICD temporal dynamics in Fig. 7E (realised as a result of blindly matching the Dll1 profiles from the sectioned dataset and the explant data set) is a prediction of the algorithm. Because *Lfng* is a known transcriptional target of Notch signalling (Morales et al., 2002; Cole et al., 2002), NICD production would be expected to directly lead to activation of *Lfng* transcription. Thus, these two time series are precisely in the order one would expect, providing strong support for the time-ordering mechanism.

The observation that both Notch1 and Dll1 are expressed in a cyclical manner at the protein level, tightly coupled with the cyclical appearance of both NICD and nascent transcripts of the Notch target gene *Lfng*, provides insight into how intracellular oscillations are coordinated, in the form of spatiotemporal waves of expression, across the PSM. These data highlight the fact that Dll1 and/or Notch1 protein is periodically cleared from the caudal PSM. Thus, receptor and ligand (known to be required for synchronisation of oscillations) are probably only available to communicate intercellularly for short pulses of time, thereby contributing to the mechanism by which activation and production of clock gene expression is pulsatile and synchronised between neighbours. These targets include *Lfng* and Notch1 itself, whereby receptor levels are replenished and a new wave of activity is initiated (Fig. 7F).

It is clear that the baseline level of Notch1 is greater rostrally than caudally. However, in isolation from the high rostral expression, oscillations of Notch1 in the caudal PSM are obvious; it is very apparent from Fig. 7D and E that fluctuations in the caudal region are large compared with the mean level and are comparable to the order of magnitude recently reported for Her1 in the zebrafish PSM (Soroldoni et al., 2014). Thus, these fluctuations are likely to be biologically relevant, given that this pathway is exquisitely sensitive to levels of both receptor and ligand. Further analysis is required to evaluate the precise location of receptor and ligand at the cell membrane and their relative levels, which could determine their implication in trans-activation or cis-inhibition of Notch signalling (Sprinzak et al., 2010).

In conclusion, our results provide the first indication of a potential mechanism by which neighbouring cells in the PSM might communicate and coordinate phase differences in a periodic manner. This opens a new area of investigation that will contribute to our understanding of the nature of this crucial mechanism both within the PSM and potentially in other biological systems that rely on dynamic spatiotemporal gene expression patterns.

## MATERIALS AND METHODS

### Cloning of intronic probes

Primers were designed from Ensembl (www.ensembl.org) to correspond to intronic sequences for each target gene. The primers were used to perform PCRs to amplify target sequences that were then cloned into the pGEM-T Easy vector (Promega). The sequences used are listed in supplementary material Table S1.

### Mouse culture

Wild-type CD1 mouse (*Mus musculus)* embryos were obtained from timed-mated pregnant females at 10.5 days postcoitum (dpc). Explants were prepared as described previously (Dale et al., 2006). In short, the tail (including the PSM and the last 2-3 formed somite pairs) was bisected to generate two identical half explants. For fix and culture, one explant was fixed immediately, whereas the other was cultured for half an oscillation cycle before being fixed. For inhibitor assays, one explant was cultured for 3-4 h (to cover at least one full oscillation cycle) in the presence of the reagent of choice, whereas the other was cultured for the same time in control medium. The reagents used were the Notch pathway inhibitor LY411575, Wnt pathway inhibitors pyrvinium pamoate (Sigma-Aldrich) and XAV939 (Tocris Bioscience) or the corresponding control, dimethylsulphoxide (DMSO, Sigma). LY411575 specifically inhibits Notch activation by preventing liberation of the intracellular domain of the Notch receptor (Ferjentsik et al., 2009). XAV939 inhibits Wnt signalling by inhibiting tankyrase enzymes that degrade Axin (Huang et al., 2009). Pyrvinium binds to casein kinase 1 and selectively potentiates casein kinase 1α, which inhibits Wnt signalling (Thorne et al., 2010). Titration assays were performed to establish the lowest concentration that abolished the expression of control target genes of the Notch and Wnt pathway, respectively. The toxicity of the treatments was determined by using the TUNEL assay (supplementary material methods). Experiments were conducted in strict adherence to the Animals (Scientific Procedures) Act of 1986 and UK Home Office Codes of Practice for use of animals in scientific procedures.

### Chick culture

Fertilised chick (*Gallus gallus*) embryos from Winter Farm, Cambridge, UK, were incubated for approximately 40 h at 38°C, 5% CO_2_ to yield embryos between HH stages 8-13. Explants were prepared as described previously (Palmeirim et al., 1997). Drug assays lasted 3 h.

### *In situ* hybridisation

*In situ* hybridisations utilising exonic and intronic probes were performed as described previously (Gibb et al., 2009; Ferjentsik et al., 2009). Probes were prepared as described previously: *Dll1* (Bettenhausen et al., 1995), *Notch1* (Lindsell et al., 1996), *Lfng* (Forsberg et al., 1998), *Lfng(i)* (Morales et al., 2002), *Snail1* (Dale et al., 2006), *mHes7* (Niwa et al., 2007). Probes are exonic, except when used to detect pre-RNAs.

### FISH

FISH for exonic probes was performed as described previously (Denkers et al., 2004). Samples were permeabilised in 2% Triton X-100 in PBS for 1 h at room temperature before counterstaining in 1 mg/ml DAPI in PBS for two nights at 4°C. Samples were mounted onto slides with a 0.12 mm spacer (Grace Bio-Labs) in glycerol-based mountant containing 0.5% p-phenylenediamine. All images were obtained using a Zeiss 710 confocal microscope and ×40 oil-immersion objective, with optical sections taken at 4 µm intervals. Multiple images from each *z* plane were stitched to form a complete overview image using Zen 2011/2012 software. Maximum intensity projections were generated in Volocity.

### qRT-PCR and statistical analysis

Mouse explants were prepared for fix and culture. The cDNA was produced from each caudal half PSM using standard techniques. qRT-PCR was accomplished in the presence of SYBR Green Mastermix (Primer Design), and reactions were measured in a Mastercycler ep realplex (Eppendorf) with the following cycling conditions: 95°C for 10 min, 45 cycles at 95°C for 15 s and 60°C for 60 s. qPCR was performed using primers described previously [*Dll1* and *Gapdh* (Kobayashi et al., 2009); *Hes7* and β-actin (Ferjentsik et al., 2009); *Notch1* (Kaltezioti et al., 2010); see supplementary material Table S2 for sequences]. Normalisation was performed against *Gapdh* using the Pfaffl equation (Pfaffl, 2001), and Fisher's F-test, as well as ANOVA, was used to measure differences between all sample variances carried out in R (R Core Team, 2013; http://www.R-project.org/).

### Whole-mount immunohistochemistry

E10.5 tails were fixed for 1 h in 4% paraformaldehyde in PBS at room temperature before tissue permeabilisation in 2% Triton X-100 in PBS for 1 h at room temperature with agitation. Samples were blocked in 2% BSA, 10% normal goat serum (NGS) in PBST for 4-12 h in the dark. Incubation with both primary antibodies (Cell Signaling Technology, anti-Notch1, 1:25; anti-Dll1, 1:50) was conducted in working buffer (0.2% BSA, 0.3% NGS, 0.2% Triton X-100 in PBS) for 72-120 h at 4°C. Samples were washed twice for 5 min in PBS and three times for 10 min in 2% Triton X-100 in PBS before the addition of Alexa Fluor-conjugated secondary antibodies in working buffer containing 20 µg/ml Hoechst 33342. Samples were incubated with secondary antibody for 72 h at 4°C and washed thoroughly in PBS before mounting onto slides with a 0.12 mm spacer (Grace Bio-Labs) in Tris-buffered glycerol-based mountant containing 0.5% p-phenylenediamine as an anti-fade. All images were obtained using a Zeiss 710 confocal microscope.

### Paraffin sections and double immunohistochemistry

Prior to double immunohistochemistry, E10.5 tails were fixed, sectioned to generate sagittal paraffin sections (7 µm) and antigen was retrieved. Endogenous horseradish peroxidase (HRP) activity in the sections was quenched using 1% hydrogen peroxide. Primary antibody incubation for Notch1 (purified mouse anti-mouse Notch1; clone mN1A Cell Signaling Technology; 1:20), Dll1 [rat anti-Dll1 monoclonal antibody (mAb); PGPM-1F9; kindly provided by E. Kremmer (Geffers et al., 2007); 1:50] and NICD [cleaved Notch1 (Val1744); D3B8; rabbit mAb Cell Signaling Technology; 1:200] was conducted in 10% NGS in PBST for 24-72 h at 4°C. Dll1 and Notch1 antibody binding was detected using Alexa Fluor-conjugated secondary antibodies, whereas NICD signal amplification and detection were performed using a TSA-Cyanine 3 system (PerkinElmer). Samples were co-stained with DAPI (1 mg/ml in PBS), mounted in Prolong Gold and imaged on a Zeiss 710 confocal microscope. We performed the following controls: no primary antibody, no primary or secondary antibodies, each antibody separately in single immunohistochemistry. See supplementary material Table S3 for details of antibodies.

### Image analysis and quantification of expression levels

Following confocal analysis, regions of interest within the PSM in which to quantify expression levels were selected by defining a rectangular domain of tissue with axes parallel to the rostro-caudal and medial-lateral axes of the sample. To remove noise and background signal, fluorescent images were systematically background subtracted and thresholded to the level of no primary control prior to subsequent quantification. In order to control for natural variation in tissue size and to permit cross-sample comparisons, three quantities were defined in each sample: an origin, an axis and unit length. The origin was defined to be the caudal-most point in the PSM, the axis to lie parallel to the rostro-caudal axis and the unit length to be the distance between the last-formed pair of somites. The fluorescence intensity was averaged and calculated as a function of position along the rostro-caudal axis. Intensity plots were normalised such that the minimum and maximum values obtained in the axial profiles were zero and one, respectively. A similar procedure was followed for paraffin sections.

To characterise spatiotemporal patterns of Dll1 and Notch1 in one half explant, we analysed the expression of a positive control [*Lfng(i)* or NICD] in the contralateral explant to time-order a given set of tissue samples (explants or sections, respectively), thus allowing the expression patterns of Dll1 and Notch1 to be studied. We note that the experiment is blind to the stage of the pattern; hence, the time-ordered samples can be assumed to be uniformly distributed throughout the somitogenesis clock cycle. Hence, we used the time ordering as a proxy for embryonic time.

Given a time-ordered set of tissue samples, we used a kymograph, with position along the rostro-caudal axis and time represented on the *x* and *y* axes, respectively, and averaged fluorescence intensity represented by colour, to analyse spatiotemporal dynamics. We stress that rows of the kymographs represent data from individual explants. An averaging filter was applied to kymographs to aid visualisation. All image analysis was performed in MATLAB.

### Western blot analysis

Caudal PSM explants or pooled samples of nine PSM explants were lysed (50 mM Tris-HCl pH 7.4, 0.27 M sucrose, 1 mM sodium orthovanadate pH 10, 1 mM EDTA, 1 mM EGTA, 10 mM sodium β-glycerophosphate, 50 mM NaF, 1% Triton X-100, 0.1% β-mercaptoethanol) and centrifuged for 10 min at 4°C, 10,000 RPM xxxx ***g***. Samples were then separated by SDS-PAGE and transferred to nitrocellulose membranes (Whatman) and were treated overnight with mouse anti-Notch1 antibody (BD Bioscienes), rat anti-Dll1 (E. Kremmer, GmBH, Munich, Germany), rabbit anti-NICD antibody (Cell Signaling Technology) or mouse anti-α-tubulin antibody (Abcam) in 5% milk in TBST; followed by secondary antibody (conjugated to HRP) in 5% milk in TBST and standard ECL detection (Pierce). SuperSignal West Femto (Pierce) was also used to detect very low levels of protein.

### Time-ordering embryos

We developed an algorithm that facilitates the identification of spatiotemporal expression patterns from an array of static images of different embryos at the same developmental stage. Consider a set of *N* dissected embryos labelled *i*={1,…,*N*}, each with a normalised intensity profile for a positive control, as defined above, given by the function *f*(*i, x*), where *x* represents spatial position along the axis, *i* is the index for a given embryo and *f* is the average intensity at position *x* for the *i*th embryo. Each embryo is associated with some (unknown) time, *t_i_*, which represents how far it has progressed through the somitogenesis clock cycle from an arbitrarily defined initial reference point. However, the set of times {*t*_1_,…,*t_N_*} are effectively randomly sampled from a uniform distribution. We achieve time ordering as follows. For a given initial (random) ordering, we construct a space-time expression plot, also known as a kymograph, where the *x* axis represents the position along the vertebrate axis, the *y* axis represents a proxy for time and intensity levels are represented by colour. Our goal is to reorder the individual axis profiles such that we obtain a smooth kymograph (which represents the known propagating wave). This is achieved by minimising the error function:

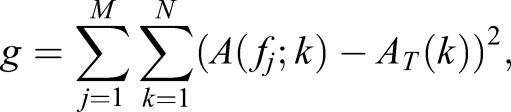


where *j* represents the position along a discretised spatial axis, *M* the number of discretised points, *A* is the normalised autocorrelation function, *f_j_* is the *j*th column of the kymograph matrix *f* and *A_T_* is a target autocorrelation chosen to enforce temporal periodicity of the pattern. For this purpose we chose:

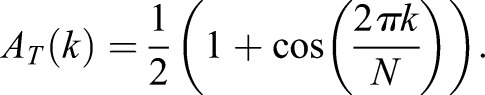


Minimisation is achieved using the Metropolis–Hastings algorithm. Given an initial random ordering, *g* is calculated. Two embryos are then randomly selected, their position in the kymograph interchanged and *g* is recalculated for the new ordering. If the reordering lowers the value of the error function, it is accepted and a new ordering is defined. If it raises the value of the error function, it is accepted with probability:

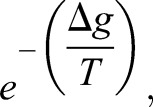


where ▵*g* is the increase in the function that occurs as a result of the interchange and *T* is an effective temperature. Otherwise the new ordering is rejected. Iterating over this procedure, we find that minimisation of *g* yields orderings in time that smoothly connect the expression profiles from the *N* embryos, and we can blindly obtain a sample ordering that yields the observed wavelike patterns of the positive control (i.e. the known clock gene).

Given a new embryo ordering that minimises and yields observed expression patterns of a known clock gene, we can therefore blindly analyse spatiotemporal expression dynamics of expression of a gene or protein of interest in the contralateral time-ordered embryo halves.

## Supplementary Material

Supplementary Material
