# Supplementary material for: Spatiotemporal oscillations of Notch1, Dll1 and NICD are coordinated across the mouse PSM
Source: Development. 2014 Dec 15;141(24):4806–16. doi: 10.1242/dev.115535 (PMC4299275; doi:10.1242/dev.115535)
Supplement: Supplementary Material [file supp_141_24_4806__index.html]

Supplementary Material 

# Spatiotemporal oscillations of Notch1, Dll1 and NICD are coordinated across the mouse PSM

## DEV115535 Supplementary Material

**Files in this Data Supplement:**

- Supplementary Material
