# Supplementary material for: Spatiotemporal oscillations of Notch1, Dll1 and NICD are coordinated across the mouse PSM
Source: Development. 2014 Dec 15;141(24):4806–16. doi: 10.1242/dev.115535 (PMC4299275; doi:10.1242/dev.115535)
Supplement: Supplementary Material [file supp_141.24.4806_DEV115535supp.pdf]

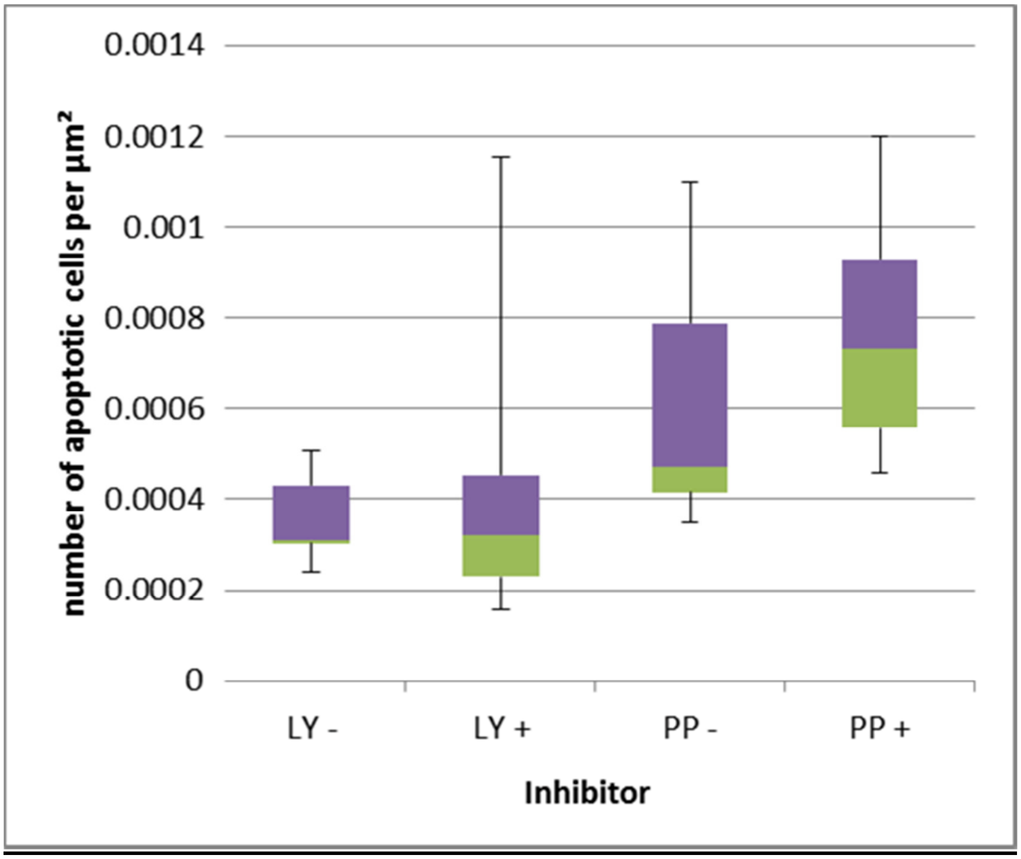

Supplementary Figure 1. Small molecular inhibitors do not induce apoptosis in mouse PSM explants after culture (see also Figure 4). Box plot showing spread of TUNEL staining measurements in mouse E10.5 PSM explants treated with LY411575 (LY) or Pyrvinium pamoate (PP) compared to untreated controls. “+” indicates treated half and “-” indicates control half (n=3 explants per drug, 9 technical replicates). p=0.289 for LY, p=0.159 for PP. The line in the box shows the median.

Supplementary Table 1: Primers used to generate anti-RNA intronic probes

Gene	Forward primer	Reverse primer
mDll1(i)	5'-GGTGCCTGACCTGCCTACAGAA-3'	5'-GAGACTCCCCAGTATCACCAGGTGG-3'
mDll3(i)	5'-GTGCCCCAGAGCCTTTACTACCC-3'	5'-CGGTGGTCAGGGAAGCTCACTG-3'
mNotch1(i)	5'-CACGCTTTGGGTAGATAGGAG-3'	5'-GGAAGGTCAGAAGAGAGAGG-3'
cDll1(i)	5'-CGAAGCCGCAGTCGTCCTCCT-3'	5'-TTGGCTGTTTCGTAGCTC-3'
cNotch1(i)	5'-ACGGCTTCTGCGCAAACCTG-3'	5'-TGCATCCGATACCCAGGCTGC-3'

Supplementary Table 2: Primers used for quantitative RT-PCR analysis

Gene	Forward primer	Reverse primer
Dll1	5'-TCAGATAACCCTGACGGAGGC-3'	5'-AGGTAAGAGTTGCCGAGGTCC-3'
Notch1	5'-GCCGCAAGAGG CTTGAGAT-3'	5'-GGAGTCCTGGCATCGTTGG-3'
Hes7	5'-GAAGCCGTTGGTGGAGAAG-3'	5'-GGCTTCGCTCCCTCAAGTAG-3'
β-actin	5'-GGCTGTATTCCCCTCCATCG-3'	5'-CCAGTTGGTAACAATGCCATGT-3'
GAPDH	5'-ATGAATACGGCTACAGCAACAGG-3'	5'- CTCTTGCTCAGTGTCCTTGCTG-3'

Supplementary Table 3: Details of antibodies used in immunohistochemistry

Antibody	Species	Dilution used in immunochemistry		Origin of antibody
		Sections	Wholemount	
anti-Notch1 (mN1A)	mouse	1:20	1:25	BD Biosciences
anti-Dll1 (PGPM-1F9)	rat	1:50	1:50	Kind gift from E.Kremmer
anti-Cleaved Notch1 (NICD) (Val1744) (D3B8)	rabbit	1:200		Cell Signalling Technologies

Supplementary Table 4: ANOVA analysis of significant differences in variance

	Df	Sum Sq.	Mean Sq.	F value	Pr(>F)	Significance
Gene	3	5.257	1.7525	13793.64	<2e-16	***
Sample	15	0.323	0.0215	169.52	<2e-16	***
Gene: Sample	45	0.278	0.0062	48.59	<2e-16	***
Gene:Sample:Fix.cult	45	0.128	0.0028	22.38	<2e-16	***
Sample:Fix.cult	15	0.053	0.0036	27.98	<2e-16	***
Gene:Fix.cult	3	0.047	0.0157	123.5	<2e-16	***
Fix.cult	1	0.021	0.0207	162.83	<2e-16	***
Residuals	251	0.032	0.0001			

Significance codes: 0 '***' 0.001 '**' 0.01 '*' 0.05 '.' 0.1 ' ' 1

Supplementary Table 5. Fisher's F-test for differences between sample variances in RT-qPCR fix and culture analysis

	Normalised to GAPDH				Normalised to β-actin			
	**Dll1**	**Notch1**	**Hes7**	**β-actin**	**Dll1**	**Notch1**	**Hes7**	**GADPH**
**Dll1**	-	0.014	0.0001229	<0.001	-	0.006904	0.0006761	<0.001
**Notch1**	-	-	0.1075	<0.001	-	-	0.4196	<0.001
**Hes7**	-	-	-	<0.001	-	-	-	<0.001

Fisher's F-test for differences between the total variance for each gene across all fix/culture caudal PSM explant pairs carried out in R and the outcomes are shown. The values in the table indicate the significance of the deviation of the relevant variance ration from unity. Values were normalised to either *mGAPDH* or *mβ-actin*

Supplemental Experimental Procedures

Apoptosis Assay

The ApopTag kit (Chemicon) was used to perform a version of the TUNEL assay on chick PSM explants with the following modifications. Samples were prepared for the assay by proteinase K treatment and fixation (4% formaldehyde in PBS, 2 mM EGTA, 0.1% glutaraldehyde) prior to washing in PBST. Equilibration buffer was then added to the explants and left for 15 min at room temperature. Incubation in working strength terminal transferase (TdT) was carried out overnight at 4 °C and the reaction stopped with stop/wash buffer by incubation at 37 °C for 40 min. Samples were then washed for a minimum of six times in TBST before heat inactivating the enzyme at 65 °C for 20 min. Samples were then incubated in blocking reagent (20% Blocking Reagent (Roche), 20% heat inactivated goat serum in TBST) for 2 h at room temperature before addition of 150mU/ml antidigoxigenin-AP antibody (Roche) and incubation overnight at 4 °C. Explants were then washed three times for 1 h in MABT (0.1 M maleic acid, 0.15 M NaCl, 10% Tween 20, pH 7.5) followed by two 10 minute washes in NTMT (100 mM NaCl, 100 mM Tris HCl pH 9.5, 50 mM MgCl2, 1% Tween 20). Samples were then incubated in NBT/BCIP colour reagent (0.027% 50 mg/ml NBT (Promega), 0.014% 50 mg/ml BCIP (Promega) in NTMT) for a minimum of 5 min at room temperature until sufficient signal had developed. Samples were then sectioned at 10μm, and imaged using a Leica DM5500B microscope using a Hamamatsu camera. Labelled cells in the PSM were counted manually using the 20x objective. The number of dots per section were counted and divided by the total area to produce a ratio of apoptosis per section. The results were then analysed by paired T-Test. Results were also shown graphically by means of a boxplot to show the spread of the data, with the box displaying the middle half of the data.
